# Artificial intelligence-directed acupuncture: a review

**DOI:** 10.1186/s13020-022-00636-1

**Published:** 2022-06-28

**Authors:** Yulin Wang, Xiuming Shi, Thomas Efferth, Dong Shang

**Affiliations:** 1 College of Pharmacy, Dalian Medical University, 9 South Lvshun Road Western Section, Dalian, 116044 People’s Republic of China; 2 Renaissance College, University of New Brunswick, 3 Bailey Drive, P.O. Box 4400, Fredericton, NB E3B 5A3 Canada; 3 Department of Pharmaceutical Biology, Institute of Pharmaceutical and Biomedical Sciences, Johannes Gutenberg University, 55128 Mainz, Germany; 4 Clinical Laboratory of Integrative Medicine, First Affiliated Hospital of Dalian Medical University, 222 Zhongshan Road, Dalian, 116011 People’s Republic of China; 5 College of Integrative Medicine, Dalian Medical University, Dalian, 116044 People’s Republic of China

**Keywords:** Artificial intelligence, Acupuncture, Machine learning, Traditional Chinese medicine

## Abstract

Acupuncture is widely used around the whole world nowadays and exhibits significant efficacy against many chronic diseases, especially in pain-related diseases. With the rapid development of artificial intelligence (AI), its implementation into acupuncture has achieved a series of significant breakthroughs in many areas of acupuncture practice, such as acupoints selection and prescription, acupuncture manipulation identification, acupuncture efficacy prediction, and so on. The paper will discuss the significant theoretical and technical achievements in AI-directed acupuncture. AI-based data mining methods uncovered crucial acupoint combinations for treating various diseases, which provide a scientific basis for acupoints prescription in clinical practice. Furthermore, the rapid development of modern TCM instruments facilitates the integration of modern medical instruments, AI techniques, and acupuncture. This integration significantly improves the quantification, objectification, and standardization of acupuncture as well as the delivery of clinical personalized acupuncture therapy. Machine learning-based clinical efficacy prediction of acupuncture can help doctors screen patients who may benefit from acupuncture treatment. However, the existing challenges require additional work for developing AI-directed acupuncture. Some include a better understanding of ancient Chinese philosophy for AI researchers, TCM acupuncture theory-based explanation of the knowledge discoveries, construction of acupuncture databases, and clinical trials for novel knowledge validation. This review aims to summarize the major contribution of AI techniques to the discovery of novel acupuncture knowledge, the improvement for acupuncture safety and efficacy, the development and inheritance of acupuncture, and the major challenges for the further development of AI-directed acupuncture. The development of acupuncture can progress with the help of AI.

## Introduction

Acupuncture represents an integral component of traditional Chinese medicine (TCM) that has been widely used in China for the past three thousand years [1]. Acupuncture treatment uses hair-thin needles to trigger the meridians by stimulating acupoints to restore the flow of *Qi* and the equilibrium within the human body [2–4]. Today, even in western countries, acupuncture is an appealing alternative treatment for ameliorating chronic diseases, such as asthma, migraine, stroke complications, menstrual issues, knee and back pain, chemo-induced nausea, vomiting, and hot-flash [1]. Many patients with chronic diseases turn to acupuncture treatments if conventional practices do not seem to work for them [5–7].

Traditional acupuncture derives from ancient Chinese philosophy, which is rich with historical and cultural values [4, 8]. Due to the rapid development of TCM research, acupuncture has advanced with modern science and technology, including artificial intelligence (AI) [9, 10]. Many important acupoints combinations for the treatment of various diseases have been discovered from the big and complicated clinical data of acupuncture therapy through using AI-based data mining methods. Previously, the design of acupoints prescription depended on the experience of acupuncturists. Currently, AI-driven knowledge discovery provides scientific evidence for acupoints prescription in clinical practice. Apriori algorithm was widely used in this field. Furthermore, distinct acupuncture manipulation can be automatically identified by AI-based differentiate systems, in which AI-based Computer Vision technology plays a crucial role. The manipulation of young acupuncturist can be determined accurately and objectively by those systems. The systems also can professionally record the manipulation of master acupuncturist, which makes great sense for TCM inheritance. Machine learning-based models can predict the efficacy of acupuncture therapy for any specific patient and provide crucial information for acupuncture doctors to screen the patients who will benefit from acupuncture therapy. Up to now, AI-directed acupuncture has achieved significant breakthroughs in many fields. However, there are still existing challenges for developing AI-directed acupuncture, including model interpretation, novel discovery of acupuncture knowledge validation, integration of ancient Chinese philosophy and modern machine learning algorithms, development of acupuncture special instruments for signal collection, and the construction of well-designed acupuncture database.

This review summarizes the contribution of AI techniques to discover novel acupuncture knowledge, improve acupuncture safety and efficacy, and develop and investigate acupuncture theory and techniques and its challenges.

## AI-directed acupuncture point selection and prescription

Acupuncture point selection and prescription is one of the key and fundamental steps in acupuncture treatment. There are 361 acupoints located on 14 meridians within the human body [11–13]. The principle of acupoint selection and prescription derives from *Yinyang* and *Wuxing* theory, the core of ancient Chinese philosophy [4, 14–16]. To some extent, acupuncture point selection and prescription is performed similarly to TCM herbal formula prescription. First, the doctor collects patient information through TCM’s four diagnostic methods, observation, listening, questioning, and pulse analysis, based on which the syndrome is differentiated. Then, the main acupoints are selected based on the diagnosis. The final step is to prescribe the combination of acupoints. Revealing a deeper understanding of acupuncture point selection is important for developing TCM acupuncture practices among clinicians. Thus, acupuncture point selection is a prominent topic to modernize TCM. Across existing literature, TCM scholars used comparative studies using clinical trial data, in which they obtained affirmative results. However, the data from clinical trials were expensive and restricted [17, 18]. Up to now, there is still a lack of standardized acupuncture point selection protocols for many diseases. In TCM theory, each acupoint has multiple functions. Therefore, it is a complex, multi-faceted relationship between acupoints and diseases. It is a daunting task to analyze this relationship using traditional data processing methods. However, with the development of AI techniques, AI-based data mining methods have offered significant advantages for analyzing large, complicated, and imbalanced clinical data, such as the relationship between acupoints and diseases [19]. Furthermore, the well-established TCM databases, warehouses, high-quality electronic TCM records, books, and the medical literature have formed an extensive data system, which provides a platform for TCM data mining. AI-based data mining methods reveal effective acupoint combinations, the laws of acupoints compatibility, and effective acupuncture treatment plans, which have significantly improved the reliability and stability of acupuncture in clinical efficacy [20–24]. Unsupervised machine learning methods, such as association rule and clustering algorithm, are the primary tools being used in those fields [25–28]. Apriori is the most commonly used algorithm for this task. The use of AI-based data mining has led to the discovery of hidden knowledge on acupuncture point selection and prescription.

**Fig. 1:**
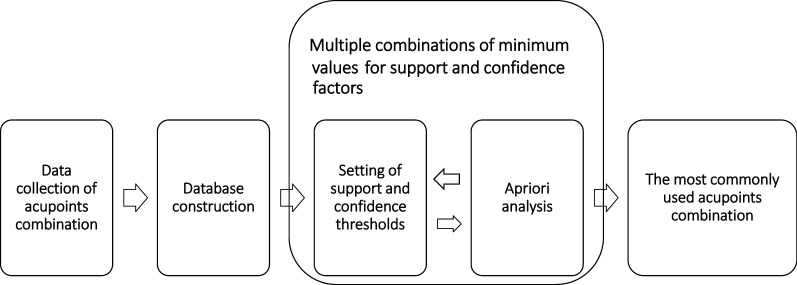
Overview of the Apriori algorithm acupoints combination data analysis procedure

Association rule is the most important data mining technique, used to explore acupuncture point combination patterns in treating diseases. The Apriori algorithm is the most commonly used method in this field, which offers insight into the acupuncture databases [22]. The principle of Apriori algorithm is to uncover the valuable association rules where the support and confidence must satisfy the minimum support and confidence confirmed by the user beforehand [29]. The first step is establishing an acupuncture points database for a particular disease to find acupuncture point combination patterns. Currently, most of the stored data on acupoints combination are in Chinese databases, such as China National Knowledge Infrastructure (CNKI), Chinese Biomedical Database (CBM), Database of Chinese sci-tech periodicals (VIP), and *Wanfang* Database. Data also exists in English databases, including Science Direct, PubMed, Web of Science, EMBASE, CENTRAL, clinicaltrials.gov., and Cochrane Library [21–23, 28]. However, the English databases are not the primary data sources for the reason that most of the studies were published in Chinese in this field and they have reached a high academic level. As Fig. 1 shows, many important Apriori model parameters were calculated out based on the well-constructed database. Support and confidence factors are the two key parameters in Apriori algorithm, based on which the frequent itemsets were generated. Support is an indicator of how frequently an acupoint appearing in all the formulas. Meanwhile, confidence is an indication of how often acupoint A appearing in the formula, given that acupoint B appears simultaneously. The choice of support and confidence thresholds will strongly influence the accuracy of Apriori algorithm. Therefore, the researchers have to test multiple combinations of minimum values for support and confidence factors during the exploration of the most commonly used acupoints combination. As reported, many important prescriptions of acupoints combination for treating a particular disease have been discovered by using Apriori algorithm.

The study conducted by Yu and his colleagues revealed that *Sanyinjiao* (SP6), *Qihai* (CV6), and *Guanyuan* (CV4) are the most commonly used acupoints combination for treating dysmenorrhea [26, 27]. Similar studies found that *Dazhui* (GV14)—*Xinshu* (BL15) *Fenglong* (ST40) is the most commonly used acupoints combination for the treatment of epilepsy [26, 28]. *Jiache* (ST6)*, Dicang* (ST4)*, Lieque* (LU7)*, Lianquan* (CV23), and *Shuigou* (GV26) are the top 5 frequently selected acupoints for neurogenic dysphagia. The meridians of bladder, stomach, liver, and spleen were the appropriate meridians for the treatment of cancer pain, with *Zusanli* (ST36)*, Neiguan* (PC6)*, Taichong* (LR3)*, Hegu* (LI4), and *Sanyinjiao* (SP6) as the core acupoints [23]. The top 10 frequently selected acupoints were *Zhongwan* (CV12)*, Zusanli* (ST36)*, Neiguan* (PC6)*, Tianshu* (ST25)*, Weishu* (BL21)*, Pishu* (BL20)*, Shenshu* (BL23)*, Sanyinjiao* (SP6)*, Ganshu* (BL18)*,* and *Liangmen* (ST21) for the treatment of diabetic gastroparesis, with *Shenshu* (BL23)*, Ganshu* (BL18)*, Sanyinjiao* (SP6)*, Pishu* (BL20), and *Neiguan* (PC6) as the core acupoints [22]. *Neiguan* (PC6)*, Taiyuan* (LU9), and *Zusanli* (ST36) were the top three frequently used acupoints for chronic stable angina pectoris (CSAP), while the pericardial meridian, lung meridian, and heart meridian were the appropriate meridians. Furthermore, *Taiyuan* (LU9) combined with *Neiguan* (PC6) was the most frequently used acupoint combination, whereas *Neiguan* (PC6)*, Xinshu* (BL15)*, Fenglong* (ST40), and *Tanzhong* (CV17) were the core acupoints [21]. Furthermore, there still are few acupoints combination studies that performed data mining on TCM books, such as the work of Hou and Hong [20]. The authors found indiscernible amounts of cross-connections in the Hasse diagram generated from the formal context. The abundance of cross-connections poses a barrier for researchers to discover knowledge for acupuncture treatment from the figure. Instead, they developed a novel knowledge discovery approach for analyzing the acupoints combinations in the treatment against respiratory diseases based on the theory of structural partial-ordered attribute diagram and association rule mining. All the data was collected from a TCM book, *Acupuncture Points Interpretation of 430 Kinds of Diseases* [20]. Another data mining study was performed on a TCM book, *Chinese Medical Classic*, revealed that *Hegu* (LI4)*-Jiache* (ST6) is the top acupoint combination among the 11 strong associated acupoint combinations. *Hegu* (LI4), *Jiache* (ST6), *Dicang* (ST4), *Shuigou* (GV26), and *Chengjiang* (CV24) were the core acupoints in the 9 acupoint cluster groups for treating neurogenic dysphagia in ancient times [30]. In TCM clinical practice, the main acupoints were selected based on the main syndrome of the patients according to TCM theory and the clinical experience of acupuncturist. Then, the adjuvant acupoints were selected mainly based on the type of the secondary syndromes of the patients. The adjuvant acupoints can significantly enhance the therapeutic effect of the main acupoints. Meanwhile, the adjuvant acupoints can assist the main acupoints in the treatment of concomitant symptoms. Therefore, the adjuvant acupoints were also crucial for the efficacy of acupuncture therapy. Furthermore, in addition to acupuncture, there are other acupoint stimulation therapies in TCM clinical practice. Some practices include electroacupuncture, acupressure, moxibustion, acupotomy, cupping and blood-letting. There are also Apriori analyses on the combinational data of acupuncture and electroacupuncture [31–33]. Furthermore, there is literature on AI-based studies on acupoints prescription in acupressure and auricular point therapy [34, 35]. AI-directed study on the rules of acupoints combination almost covers all the acupoints stimulation therapy.

In addition to the AI-powered data mining methods above, a special acupoint selection algorithm has been developed by Prof. Wen from Chengdu University of TCM (China) for the treatment of knee osteoarthritis (KOA). First, the data of four diagnoses and acupoint prescription were digitized by quantization. Then, the author established the relationship between the syndrome and symptoms. The algorithm established the relation between syndromes and acupoint prescription using Apriori based on a database constructed by mining literature and clinical trials. A model for the relationships among symptoms, syndromes, and acupoint prescription was then successfully developed. As discovered, this model can automatically differentiate the syndrome and recommend the corresponding acupoint-selection protocol to the TCM clinicians for treating KOA [36]. This research links more closely to clinical practice. For example, a young acupuncture doctor working in the outpatient department can input the symptoms of a patient into this model and obtain the syndromes as the primary output. Simultaneously, the model will automatically output the recommended acupoints prescription to the doctor. Therefore, the work of Professor Wen reinforces the need for training young acupuncture doctors. Furthermore, a TCM acupoint compatibility automated system has been established by Jingjing Liu, who assessed the compatibility map of TCM acupoints using the Apriori algorithm [37]. The map showed significant correlations among the acupoints for the same disease [37, 38].

Apriori algorithm exhibits a significant advantage due to its straightforward implementation. However, this method is associated with the limitation of frequently scanning in the large database for the calculation of candidate itemsets frequencies, thus, requiring more time for complicated calculations [39]. AI-directed acupuncture point selection and prescription significantly contributed to the discovery of novel TCM knowledge, which provides a crucial step towards developing TCM acupuncture theory. The technology is a novel research methodology to address a host of challenges in analyzing acupoints combination. However, studying the rules of acupoints prescription is not just a mathematical analysis. Many clinical data, such as therapeutic efficacy, patient demographic features, disease stratification and concomitant diseases, are vital for the reliability of the results, which should be collected and analyzed. However, clinical data were often completely or partially ignored in research. Therefore, it is imperative to integrate the association rule technique and the TCM domain knowledge. The ancient TCM books are directly copied, which will cause an overabundance of acupoints prescriptions. The best approach to addressing this issue is by having knowledgeable acupuncturists delete “meaningless acupoints” during data processing. TCM acupuncture doctors design the therapeutic protocol based on the syndromes of the patient, where there may be different treatments for the same disease. Therefore, syndromes must be differentiated in TCM clinical trials, which means only the same disease with the same syndromes can be classified in the same group. This is a key point for knowledge discovery on acupoints prescription from clinical trial data. Meanwhile, there is still much to be done. Some areas include constructing acupuncture-specific databases, clinical trials for the efficacy validation on novel acupoint combinations and the recommended acupoint prescriptions, TCM acupuncture theory-based explanation on the novel discovered acupoint combination, to name a few. AI-directed acupuncture point selection and prescription is a promising field in the future.

## AI and acupuncture manipulation

Acupuncture manipulation is a major component of acupuncture therapy, which is crucial for its therapeutic effects [40–42]. Acupuncture manipulation mainly refers to the twirling-rotating and lifting-thrusting motions of the needle at the desired acupuncture point with controlled depth and frequency [40, 43]. As reported, the nerve excitability, local oxygen tension, chemical concentration, and temperature around the same acupoint appear to have variations with different acupuncture manipulations [40, 42, 44]. Therefore, the quantification and standardization of acupuncture manipulation, such as the exertion strength, duration, and direction of acupuncture, is crucial for acupuncture clinical efficacy, as well as AI-directed acupuncture manipulation [41, 45–47]. Association rule algorithm and clustering algorithm are the main AI approaches widely used in this fields [26, 45, 47].

Many vital breakthroughs have been achieved with the help of acupuncture manipulation determination instruments. An instrument developed by the Shanghai University of Traditional Chinese Medicine can accurately determine the parameters of acupuncture manipulation and sort them out during therapy [47–49]. Currently, a standard TCM acupuncture manipulation database has been established based on the parameter data of various acupuncture manipulations collected by the instrument [45, 48]. The database serves as a crucial resource for the AI-assisted research on acupuncture manipulation. Tang and his colleagues used the instrument to develop a quantification and classification model for lifting-thrusting manipulations using a self-organizing neural network feature map [46]. The manipulation of the acupuncturist can be identified automatically and objectively by this model. Other clustering analysis studies on the manipulation parameters have also identified acupuncture manipulation in this instrument [26, 47]. All the findings above enhance the quantification, objectification, and standardization of acupuncture manipulation.

As reported by Gou, an acupuncture manipulation identification system has been developed using Fuzzy C-Means. This work was conducted using a tactile sensor, through which the signals for various acupuncture manipulations were collected. Additionally, Gou indicates a window segmentation method for tactile signal and extracts the time domain features of the window. This system can effectively identify the four basic acupuncture manipulations, i.e., reinforcing or reducing by twirling and rotating, as well as reinforcing or reducing by lifting and thrusting [41]. Therefore, this work is conducive for the objectification, quantification, and standardization of acupuncture manipulations. Another computer vision-based identification system for acupuncture manipulation was developed based on the hybrid of convolutional neural network (CNN) and long-short term memory (LSTM) neural network, which were used to extract the spatial–temporal features of video frame sequences for the further classification by softmax classifier. This system can successfully differentiate “twirling” and “lifting and thrusting” manipulations in 200 videos with the training and verification accuracy of 95.4% and 95.3%, respectively [50]. There are other acupuncture manipulation identification models based on signals can be collected by medical instruments, such as the electroencephalogram (EEG). Yu et al. proposed an EEG signal-based classification framework for different acupuncture manipulations. Within their study, the topological features of graph theory were extracted and used as the inputs for support vector machine (SVM) classifiers. This model can achieve the highest accuracy of 92.14% [40].

The rapid development of TCM instruments enables to collect digital signals of various acupuncture manipulation, improving the AI-based research in this field [51–53]. The combination of AI-directed acupuncture manipulation with VR (virtual reality) techniques help train young acupuncturists in China [38]. The acupuncture manipulation techniques of acupuncture masters are brilliant cultural practices. The objective and accurate records on acupuncture manipulation techniques are evident for TCM inheritance. AI-based research on acupuncture manipulation has brought many scientific breakthroughs in TCM modernization. Currently, some important data on acupuncture manipulation still cannot yet be recorded accurately, such as the exertion strength, duration, and direction of acupuncture. Therefore, those crucial data were omitted in the researches. However, with the unprecedented integration of modern engineering and TCM, an increase in TCM acupuncture recording and measuring instruments will be developed and used in clinical acupuncture practice. As a result, AI-based research on acupuncture manipulation will enter the era of big data in the future, further supporting the field’s progress. The significance of the research in this field is the contribution to the objectification and standardization of acupuncture manipulation, which will significantly improve the safety and efficacy of clinical acupuncture therapy.

**Fig. 2:**
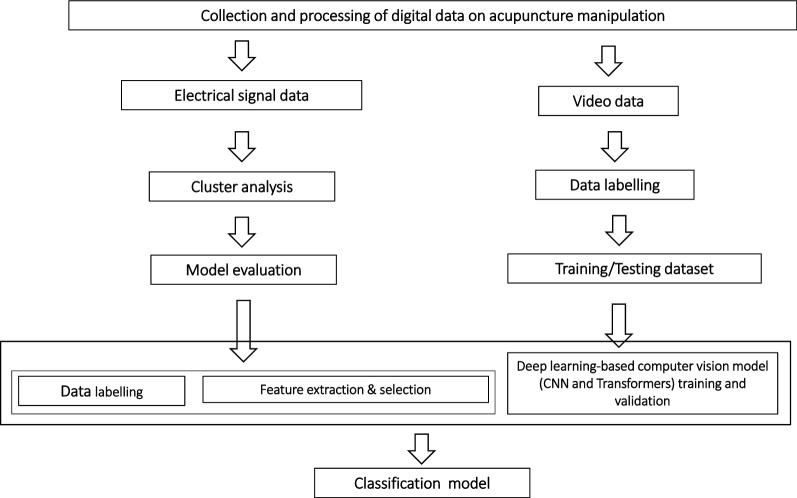
Flowchart of models for acupuncture manipulation classification

In AI-directed acupuncture manipulation identification, cluster analysis methods, such as self-organizing neural network feature maps and Fuzzy C-Means, were mainly used in electrical signal data-based studies (see Fig. 2). The principle of clustering is to group objects in a class that has a very similar resemblance to other objects in the same class and minimize the similarities of the objects from different groups. As a powerful learning technique, clustering exhibits a significant advantage as it efficiently analyzes big data. Then, acupuncturists label the clustered data to create a predictive model for the classification of tested samples. However, the performance of cluster analysis heavily depends on expert feature engineering. Therefore, the integrating machine learning and acupuncture domain knowledge presents a limitation for clustering research. With the development of calculation capacity, deep learning-based computer vision algorithms, such as CNN and Transformers, should be the best methods for analyzing video data. CNN is an ideal image feature extractor, which can extract the local features from high-resolution feature maps and combine them into more abstract low-resolution feature maps. There are several stacked layers in CNN, where the responses from the previous layer are convoluted with a filter bank and activated by a differentiable non-linearity. Deep learning-based methods can perform the work of feature learning effectively and efficiently. However, these methods typically depend on high-level calculation capacity and a large amount of labelled data.

## AI in the prediction of acupuncture efficacy

**Fig. 3:**
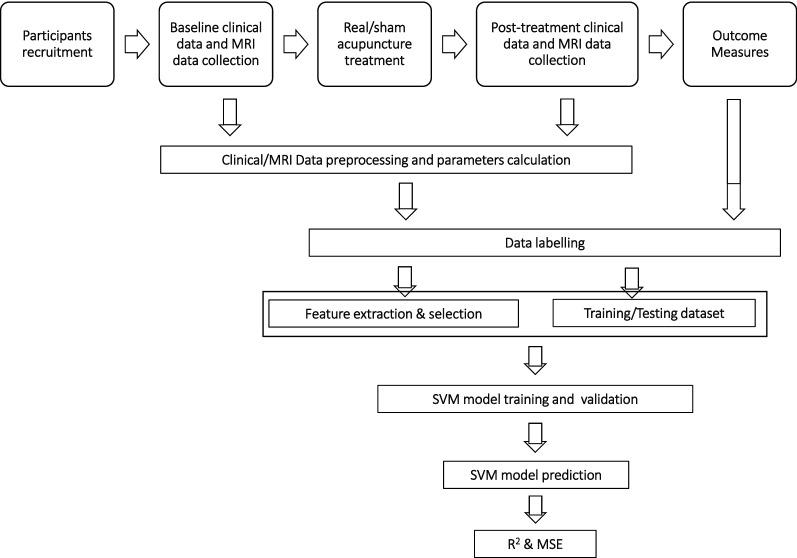
Flowchart of SVM algorithm for acupuncture efficacy prediction

Neuroimaging-based clinical efficacy prediction of acupuncture using machine learning has been an attractive research area in the field of AI-directed acupuncture, which brings a novel and promising approach to understanding the efficacy of acupuncture treatment at the individual level [54]. Neuroimaging machine learning can learn a function based on the patient clinical examination data and acupuncture efficacy. A physician can predict the efficacy of acupuncture treatment through inputting patient data of clinical examination into the function. Functional magnetic resonance imaging (fMRI) has become the most popular method for imaging brain function. The imaging method provides almost all the data for the development of the efficacy prediction model of acupuncture therapy, which primarily includes amplitude of low frequency fluctuation (ALFF), functional connectivity (FC), and regional homogeneity (ReHo) [55, 56]. SVM the most widely used prediction algorithm for acupuncture efficacy. Figure 3 shows the flowchart of SVM algorithm for acupuncture efficacy prediction. AI-based acupuncture efficacy prediction has achieved a sufficient accuracy [21, 57, 58]. The work in this field can help us screen patients who may benefit from acupuncture treatment and provide crucial information for clinical personalized acupuncture therapy [59].

Professor Liu presented the first research in AI-based acupuncture efficacy prediction, which was on migraine without aura (MwoA) [26, 57]. As reported, nearly all research in this field focuses on pain-related diseases [56, 58–63]. Up to now, MwoA, a chronic neurological disorder characterized by headache, is the most extensively studied disease, with support vector machine (SVM) as the most popularly used algorithm [56, 61–64]. As reported, SVM is more reliable when the number of patients included in the study is less than 200 [65]. The research reveals that pre-treatment brain structure can predict acupuncture efficacy prediction in MwoA [57]. In the study by Professor Liu, the researchers developed an AI model to predict the outcomes of 8-week sham acupuncture treatment in MwoA patients using SVM. The author collected all the neuroimaging data via magnetic resonance imaging before acupuncture treatment. The combined features of diffusion measures from vertices along the pathways of the medial prefrontal cortex-amygdala located in the external capsule and ACC/mPFC were used as the biomarker to predict the efficacy of acupuncture treatment. The model achieved an accuracy of 84.0% [57]. Then, analogous investigations were conducted on MwoA patients who have received acupuncture treatment. Yang also collected pre-treatment neuroimaging data from fMRI, which helped identify clusters of significant voxels. Then, the average gray matter (GM) volume across the voxels in each cluster was extracted as the initial feature. The least absolute shrinkage and selection operator (LASSO) method refined the initial features into fewer and significant features, which helped construct the SVM model. The results reveal that the model possesses a high degree of precision (sensitivity 73%, specificity 85%, accuracy 83%, and DSC 75%). Meanwhile, the area under the receiver operating characteristic curve was 0.7871 [61]. As reported, Yin and his colleagues extracted the predicting features using the advanced multivariate pattern analysis from the z-transformed amplitude of low-frequency fluctuation (zALFF) maps of 40 patients with MWoA and 40 healthy subjects, who have received a 4-week acupuncture treatment. The extracted features mainly included the zALFF value of the foci in the bilateral middle occipital gyrus, right fusiform gyrus, left insula, and left superior cerebellum. Then, the construction of support vector regression models helped discriminate patients with MWoA from healthy subjects, which indicates an accuracy higher than 70%. Furthermore, an efficacy prediction model using SVM based on the features of the zALFF value of the clusters in the right and left middle occipital gyrus can effectively predict the relief of headache intensity (R^2^ = 0.38 ± 0.059, mean squared error = 2.626 ± 0.325), and frequency of migraine attacks (R^2^ = 0.284 ± 0.072, mean squared error = 20.535 ± 2.701) [63].

As the most widely used prediction algorithm for acupuncture efficacy, SVM was also applied in the research of other pain-related diseases. Tu and et al. developed an SVM-based model to predict the efficacy of 4-week real and sham acupuncture treatment for chronic low back pain (cLBP), which used multivariate resting-state functional connectivity within and across four networks-default mode network (DMN), salience network (SN), chief executive network (CEN), and sensorimotor network (SMN). The prediction model obtained a squared correlation of 34.3 ± 5.5% (*P* = 0.033) between actual and predicted treatment responses [58]. Yu et al. used an SVM-based multivariate pattern analyses (MVPA) approach to predict the acupuncture treatment efficacy in patients with primary dysmenorrhea (PDM). Pain-related functional connectivity (FC) matrices were constructed at the baseline and post-treatment periods. The results of MVPA show that the FC patterns of descending pain modulatory system (DPMS), SMN, SN and SMN, and the SN and DMN are features for the construction of the SVM model. This model can obtain a squared correlation of 0.27 (*P* = 0.002) and an MAE of 0.36 for the prediction in the visual analogue scale (VAS) change scores after treatment, while can obtain a squared correlation of 0.30 (*P* = 0.0009) and an MAE of 2.26 for the prediction in VAS change rate [55].

In addition to pain-related diseases, there are few studies on other health conditions. Yin and his colleagues developed a predictive model using SVM for the acupuncture therapy efficacy in patients with functional dyspepsia. In their study, the authors extracted the feature regions of interest with edge detection at baseline. The connectivity between the right insula and left precuneus was the most significant feature for prediction. This model achieves an accuracy of 84.9% [56].

The studies discussed show that SVM is the most widely used machine learning method in AI-based acupuncture efficacy prediction. SVM extracts significant features from the data to create a model for predicting classification by identifying an optimal separating hyperplane with maximal margins between the vectors of the two classes. The sample size is one of the key influences for selecting machine learning methods. As a traditional machine learning method, SVM exhibits excellent performance and generalization ability for small sample sizes. Furthermore, this method can map the correlation between the clinical features and predicted outcomes, thus has strong interpretability. For these reasons, SVM is the evident choice for AI-based acupuncture efficacy prediction studies. The prediction models mentioned in this paper have achieved significant performance. However, there are still many areas to explore and validate behind the mechanisms correlating between clinical features and acupuncture efficacy. As mentioned previously, the performance of SVM heavily depends on the integration of machine learning and medical domain knowledge. With the continuous data accumulation, deep learning-based methods such as CNN, PNN, SRCNN, and GAN, have the potential to be widely used in the future. The deep learning methods above can learn the features of the data perfectly and automatically, and even can generate convincing images.

## Conclusion

AI-directed acupuncture has achieved significant progress in many areas, such as objectification and standardization of acupuncture, recommendation of acupoint prescriptions, personalized acupuncture therapy, and efficacy prediction. The major challenge to the worldwide acceptance of acupuncture is the lack of an available scientific theory on therapeutic mechanisms and evidence. Meanwhile, there are still some important limitations and future works for AI-directed acupuncture, such as the collection and administration of clinical acupuncture data, well-designed acupuncture database, the integration of TCM domain knowledge and AI techniques, the explanation on novel discovered knowledge and the availability of the source code online. However, the effective integration of AI techniques, neuroimaging, and acupuncture will bring significant breakthroughs in this field. Furthermore, a new generation of acupuncturists skilled in artificial intelligence is breathing new life into the field through the perfect integration of Chinese medicine domain knowledge and AI techniques, which will significantly promote the development of AI-assisted TCM instruments, TCM acupuncture theory and evidence-based acupuncture clinical practice. AI-directed acupuncture will make meaningful contributions to the inheritance and development of TCM acupuncture.

## Data Availability

Not applicable.

## Abbreviations

ACC: Anterior cingulate cortex
AI: Artificial intelligence
ALFF: Amplitude of low-frequency fluctuation
CBM: Chinese Biomedical Database
CEN: Chief executive network
cLBP: Chronic low back pain
CNKI: China National Knowledge Infrastructure
CNN: Convolutional neural network
CSAP: Chronic stable angina pectoris
DMN: Default mode network
EEG: Electroencephalogram
FC: Functional connectivity
fMRI: Functional magnetic resonance imaging
GAN: Generative adversarial network
GM: Gray matter
KOA: Knee osteoarthritis
LASSO: Least absolute shrinkage and selection operator
LSTM: Long-short term memory neural network
mPFC: Medial prefrontal cortex
MVPA: Pattern analyses
MwoA: Migraine without aura
PDM: Primary dysmenorrhea
PNN: Probabilistic neural networks
ReHo: Regional homogeneity
SMN: Sensorimotor network
SN: Salience network
SRCNN: Super-resolution convolutional neural network
SVM: Support vector machine
TCM: Traditional Chinese medicine
VAS: Visual analog scale
VIP: Database of Chinese sci-tech periodicals
zALFF: Z-transformed amplitude of low-frequency fluctuation
